# Therapeutic experience of a pancreatic mixed serous neuroendocrine neoplasm invading peripancreatic vessels: A case report

**DOI:** 10.1097/MD.0000000000030323

**Published:** 2022-09-02

**Authors:** Zongming Zhang, Limin Liu, Youwei Li, Zhuo Liu, Chong Zhang, Yue Zhao, Mingwen Zhu, Baijiang Wan, Hai Deng, Xiyuan Xie, Kun Tian, Zhentian Guo, Haiyan Yang, Jiahong Liao, Hongyan Zhu, Lili Liu, Man Wang, Xiaoting Ma, Tiantian Liu, Niuniu Huang, Yujiao Gao, Jing Zhao, Fang Liao, Fengyuan Li, Xueting Wang, Ruijiao Yuan, Xinying Liu, Lidan Chang

**Affiliations:** a Department of General Surgery, Beijing Electric Power Hospital, State Grid Corporation of China, Capital Medical University, Beijing 100073, China; b Youwei Li, Department of Radiology, Beijing Rehabilitation Hospital, Capital Medical University, Beijing, 100144, China.

**Keywords:** abdominal CT 3D visualization reconstruction, case report, pancreatoduodenectomy, peripancreatic vessels invaded, PMSNN

## Abstract

Pancreatic mixed serous neuroendocrine neoplasm (PMSNN) is an extremely rare disease. Only a few cases on the surgical treatment of PMSNN have been reported in the literature, and it is unclear whether there is invasion of important peripancreatic vessels.

**Patient concerns:**

We report the case of a 39-year-old female patient with PMSNN accompanied by invasion of important peripancreatic vessels. She underwent surgery and achieved satisfactory recovery.

**Diagnosis:**

Abdominal enhanced CT images showed an enhanced mass with a nonenhanced cyst involving the head and body of the pancreas, which invaded important peripancreatic vessels. The lesion had been misdiagnosed and mistreated as a metastatic carcinoma before admission.

**Interventions:**

CT 3-dimensional (3D) visualization reconstruction images showed intact peripancreatic vessels. Radical pancreatoduodenectomy was successfully performed and confirmed that the main blood vessels around the pancreas were only compressed or even wrapped by the mass, but not penetrated.

**Outcomes:**

The patient recovered well and was discharged on the 19th day after surgery. Pathological examination reported the diagnosis of PMSNN with the collision type combination and the well-differentiated grade 2 pancreatic neuroendocrine tumor. She was followed up for 18 months without any abnormalities.

**Lessons:**

This case demonstrates that surgical treatment of PMSNN with invasion of peripancreatic vessels can be successful. Preoperative abdominal CT 3D visualization reconstruction is helpful in determining the degree of invasion of important peripancreatic vessels, and plays a key role in formulating an accurate surgical plan and improving patient outcome.

## 1. Introduction

Mixed serous neuroendocrine neoplasm (MSNN) is a mixed tumor containing 2 components with different pathologies,^[1]^ a serous cystic neoplasm (SCN) and a neuroendocrine neoplasm (NEN). In the 2019 World Health Organization (WHO) Digestive System Tumor Classification, NEN was divided into neuroendocrine tumor (NET) and neuroendocrine carcinoma (NEC).^[2]^ Due to the malignant potential of the NEN component, and its close relationship with Von Hippel-Lindau (VHL) syndrome, patients with MSNN usually undergo thorough systemic examinations, surgical treatment, and close clinical follow-up.^[3]^

Pancreatic mixed serous neuroendocrine tumor (PMSNN) is an extremely rare disease. It is very difficult to determine the benignity and malignancy of its NEN component before surgery. In theory, once the diagnosis of PMSNN is made, surgical treatment is preferred. However, so far only a very few cases of surgical treatment for PMSNN have been reported in the literature,^[3–5]^ and it is unclear whether there is invasion of important surrounding vessels. In addition, there are no reports on the surgical treatment of peripancreatic vessels invasion. We report the case of a 39-year-old female PMSNN patient, without obvious symptoms, with invasion of important peripancreatic vessels and the collision type combination manifestation, but not VHL syndrome.

## 2. Case report

A 39-year-old female patient was admitted to Beijing Electric Power Hospital on December 11, 2020 with a 6-mon history of the inadvertent discovery of an abdominal mass.

The study has been approved by the ethics committee of Beijing Electric Power Hospital, State Grid Corporation of China, Capital Medical University, P. R. China. The patient has provided informed consent for publication of the case.

Six months ago before admission, the patient inadvertently found a fist-sized abdominal mass, without abdominal pain, diarrhea, fever, or other obvious discomfort. She was hospitalized twice in a Beijing famous hospital. Abdominal enhanced computed tomography (CT) and magnetic resonance imaging (MRI) showed an enhanced mass invading important surrounding blood vessels in the omental sac area, with uneven metabolism on positron emission tomography (PET)-CT. Combined with a history of “left oophorectomy for ovarian borderline mucinous cystadenoma canceration (well differentiated mucinous adenocarcinoma)” 11 months previously, and no postoperative chemotherapy was administered, the metastatic carcinoma of the lesser omental sac was diagnosed. The opinion of the multidisciplinary team was “Metastatic carcinoma of the lesser omental sac has high surgical risk and poor expected effect. Radiotherapy and chemotherapy are recommended”. She was discharged after resolutely refusing radiotherapy and chemotherapy.

During the past 6 months, the abdominal mass gradually increased without obvious weight loss, fatigue or other discomfort. She had no history of smoking, drinking, or family disease, and no contact history of COVID-19 pneumonia.

**Figure 1.**
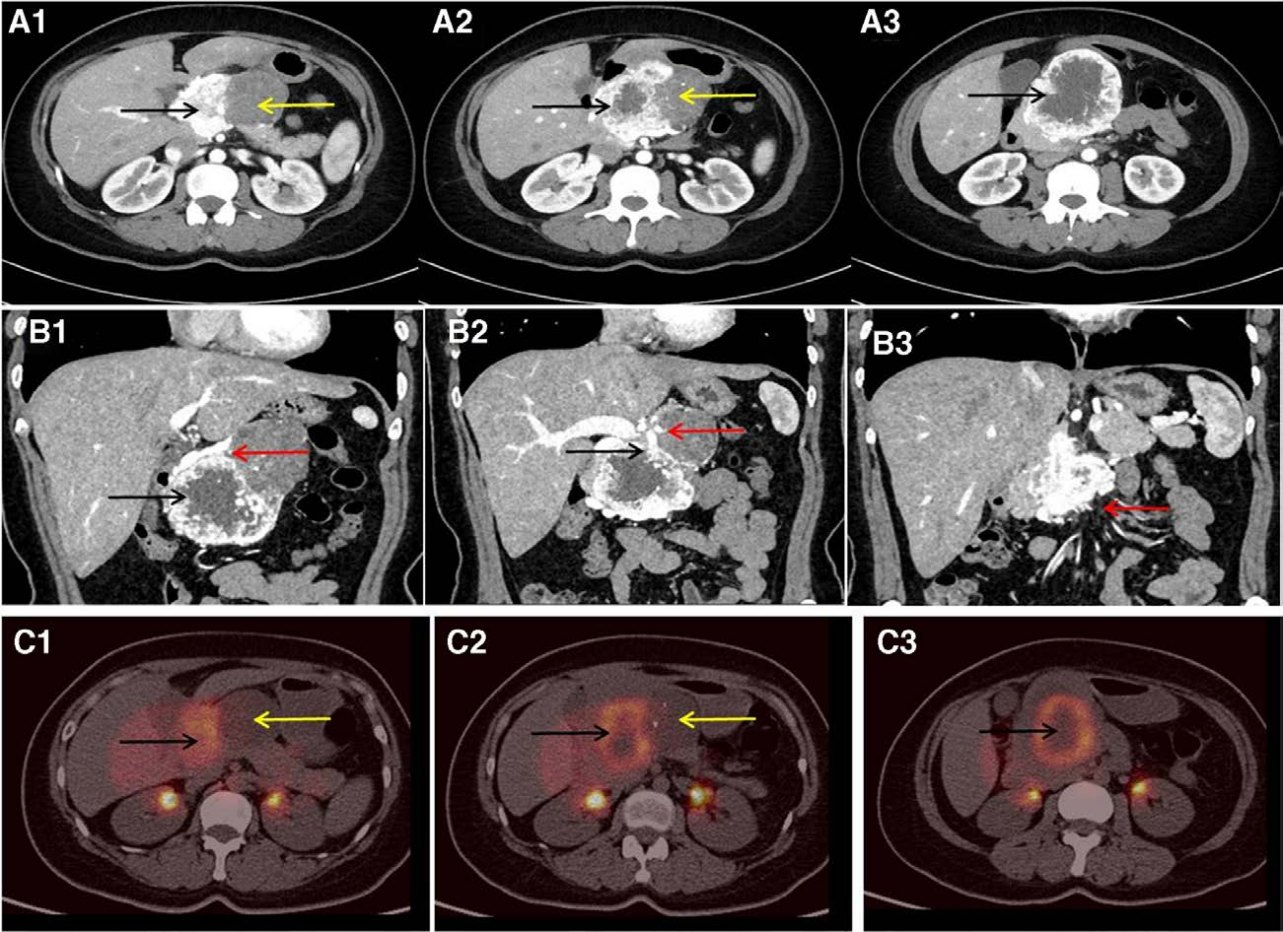
Abdominal enhanced computed tomography (CT) and PET-CT images. A1-A3: Axial enhanced CT images showed the enhanced mass (black arrow) with a nonenhanced cyst (yellow arrow) involving the head and body pancreas. B1-B3: Coronal reconstruction enhanced CT images displayed the mass (black arrow) invading its surrounding important blood vessels (red arrow); C1-C3: Axial PET-CT images revealed the unevenly fluorodeoxyglucose (FDG) uptake (black arrow) mass with nonuptake portion (yellow arrow) in lesser omental sac.

**Figure 2.**
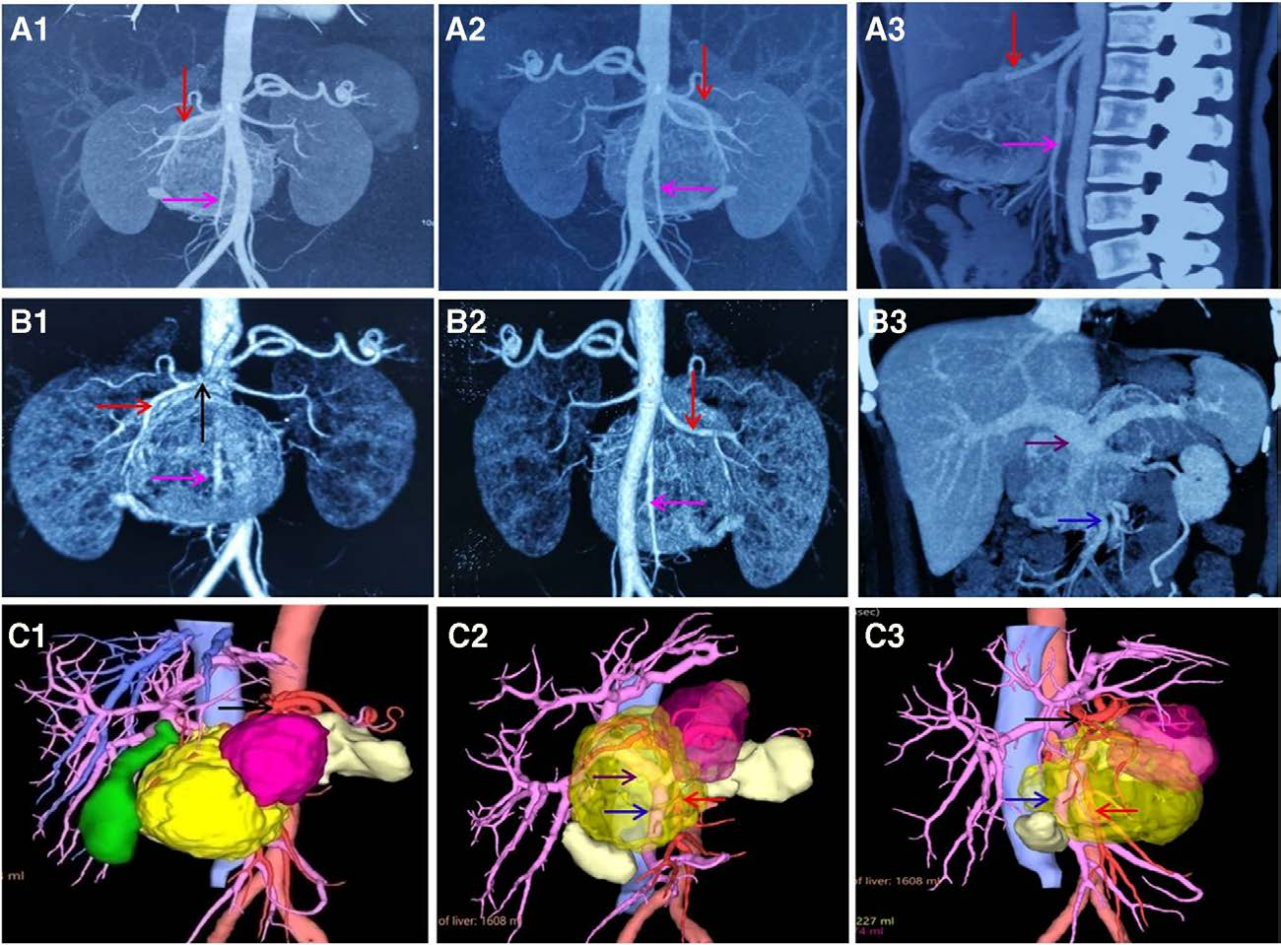
Abdominal CT 3-dimensional (3D) reconstruction images. Maximum intensity projection (MIP) images obtained in arterial phase (A1: coronal frontal view; A2: coronal dorsal view; A3: sagittal view) showed a soft tissue mass invaded the common hepatic artery (red arrow) and superior mesenteric artery (pink arrow). Coronal surface maximum intensity projection images obtained in arterial phase (B1: frontal view; B2: dorsal view) displayed the soft tissue mass (with the size of 9.4 × 8.9 × 8.6 cm) invaded the common hepatic artery (black arrow), proper hepatic artery (red arrow), and superior mesenteric artery (pink arrow). Coronal maximum intensity projection images obtained in portal phase (B3: frontal view) displayed the portal vein (purple arrow) and superior mesenteric vein (blue arrow) invaded. 3D visualization reconstruction images (C1-C3) confirmed the common hepatic artery (black arrow) invaded, but the vessel wall of the portal vein (purple arrow), superior mesenteric vein (blue arrow), and superior mesenteric artery (red arrow) kept intact.

After admission, her physical examination found that the skin and sclera did not show yellow staining, no tenderness or rebound pain was observed in the abdomen, the palpable mass under the xiphoid process of the upper abdomen was tough, smooth, inactive, and without tenderness, no ascites were observed. The laboratory examinations showed that blood sugar level was 5.84 mmol/L. Liver function tests showed that alanine aminotransferase was 22 U/L, aspartate aminotransferase was 15 U/L, total protein was 66.3 g/L, albumin was 40.1 g/L, total bilirubin was 8.85 μmol/L, direct bilirubin was 1.27 μmol/L, and γ-glutamyl transpeptidase was 60U/L. The levels of tumor markers were carbohydrate antigen 19–9 8.20 U/mL, carcinoembryonic antigen 0.828 ng/mL, pro-gastrin-releasing peptide 34.9 pg/mL, and neuron specific enolase 12.5 μg/L. The imaging examinations showed that abdominal enhanced CT images showed an enhanced mass with a nonenhanced cyst involving the head and body of the pancreas (Fig. 1A1–A3). Coronal reconstruction enhanced CT images revealed that the mass had invaded important surrounding blood vessels (Fig. 1B1–B3). PET-CT images revealed that the mass showed uneven fluorodeoxyglucose uptake with a nonuptake portion in the lesser omental sac (Fig. 1C1–C3). Abdominal CT 3-dimensional (3D) reconstruction, maximum intensity projection (MIP) images showed a soft tissue mass invading the superior mesenteric artery and inferior mesenteric artery (Fig. 2A1–A3), and coronal surface MIP images displayed the soft tissue mass (9.4 × 8.9 × 8.6 cm in size) invading the common hepatic artery, superior mesenteric artery, inferior mesenteric artery, portal vein and superior mesenteric vein (Fig. 2B1–B3). 3D visualization reconstruction images confirmed invasion of the common hepatic artery, but the vessel walls of the portal vein, superior mesenteric vein, and superior mesenteric artery were intact (Fig. 2C1–C3).

**Figure 3.**
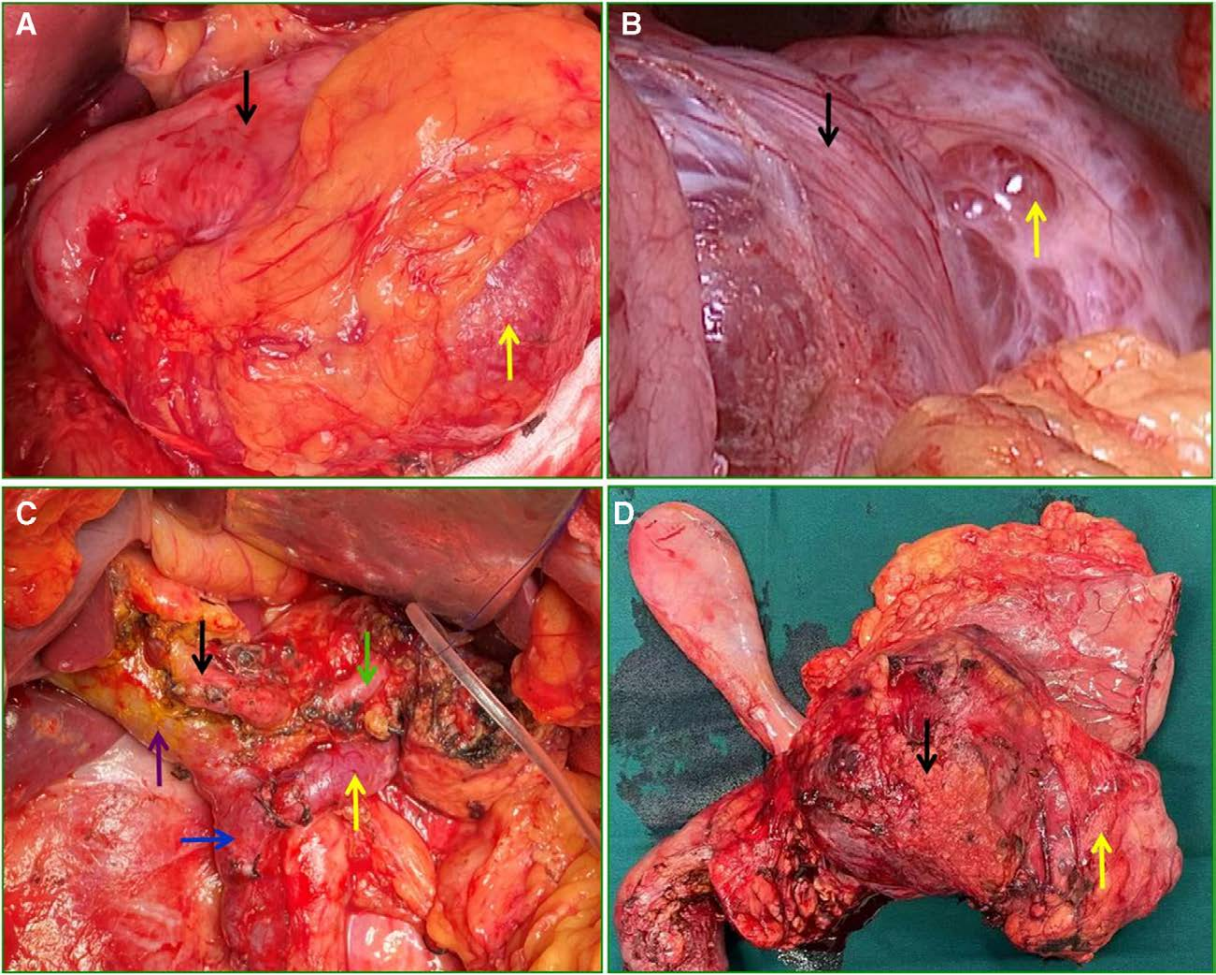
Pancreatoduodenectomy of PMSNN. A: Intraoperative findings, stomach (black arrow), tumor (yellow arrow); B: Intraoperative appearance, mainly solid part (black arrow), mainly cystic part (yellow arrow); C: Intraoperative important vascular dissociation, hepatic proper artery (black arrow), splenic artery (green arrow), splenic vein (yellow arrow), superior mesenteric vein (blue arrow), portal vein (purple arrow); D: Resected specimen following pancreatoduodenectomy, mainly solid part (black arrow), mainly cystic part (yellow arrow).

**Figure 4.**
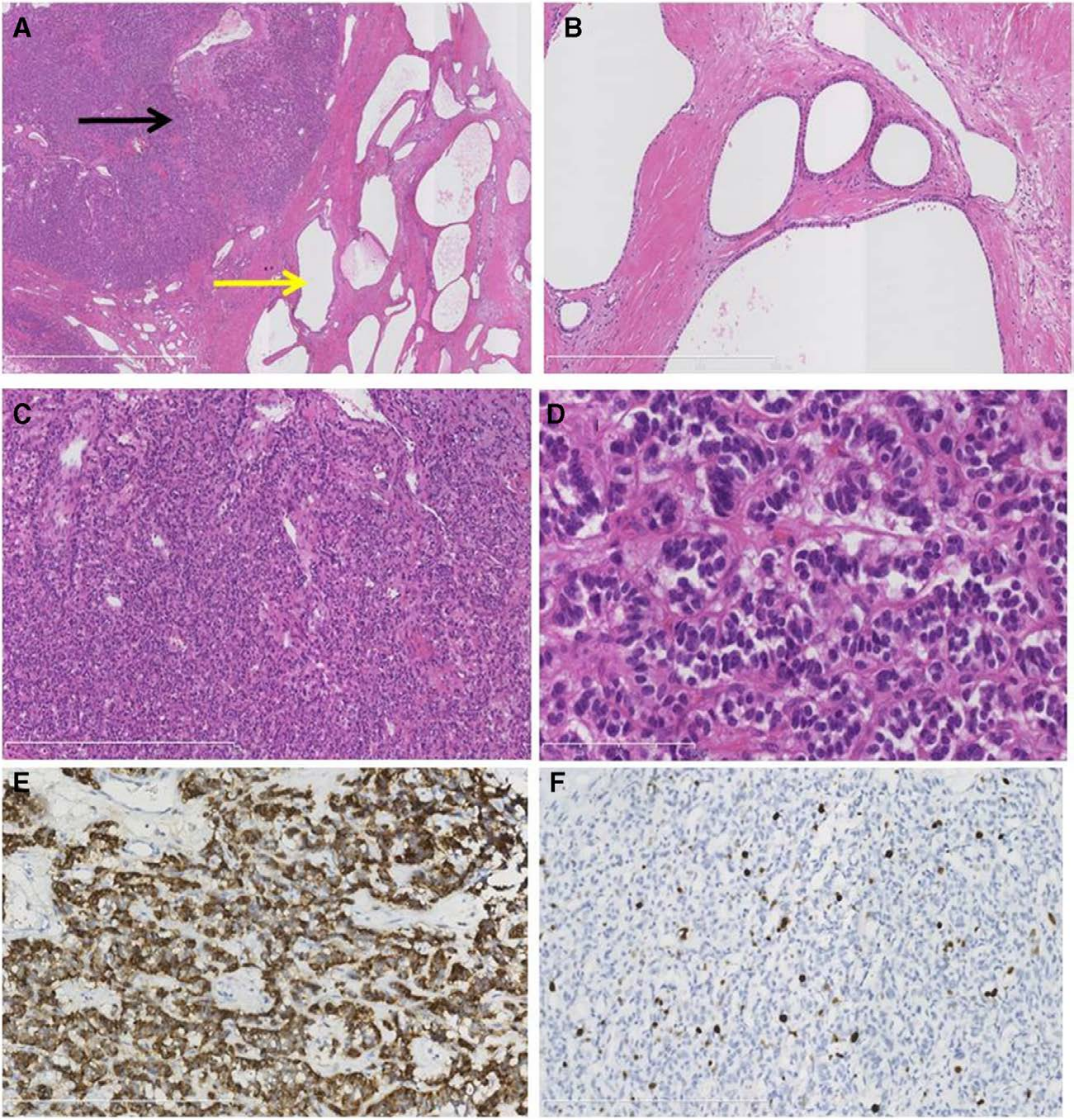
Pathological analysis of PMSNN. A: pancreatic mixed serous neuroendocrine neoplasm, containing both cystic (yellow arrow) and solid neuroendocrine (black arrow) components, with the collision type combination; B: Cystic neoplasm, the glandular duct is expanded in a cystic shape, lined with a single layer of cubic epithelium, interstitial fibrosis and sclerosis. C & D: Neuroendocrine neoplasm, cells grew in a beam or pseudoglandular arrangement, with consistent nuclear size, fine nuclear chromatin and eosinophilic or bichromatic cytoplasm, the mitotic count is 0 per 10 high power fields; HE staining (magnification, ×40 for A, ×100 for B and C, ×400 for D). E: Immunohistochemistry staining for chromogranin A, neuroendocrine tumor cells were positive (×200). F: The Ki67 labeling index of the pancreatic neuroendocrine tumor was more than 5% (×200).

Following admission, the patient was diagnosed with a malignant pancreatic tumor and extensive invasion of important surrounding blood vessels. Based on the adequate preparation of artificial blood vessels and allogeneic blood vessels, radical pancreatoduodenectomy was successfully performed on December 29, 2020 (Fig. 3D). During the operation, the tumor (approximately 12 × 8 cm in size) was found in the pancreatic head and body, and was mainly solid in the right section, mainly cystic in the left section (Fig. 3A), and smooth on the surface (Fig. 3B). It did not invade the stomach, duodenum or transverse colon. The surrounding tissues and blood vessels near the tumor were carefully stripped. The common hepatic artery, superior mesenteric artery and vein, splenic artery and vein, and portal vein were obviously compressed or wrapped by the mass, but did not penetrate the vascular walls (Fig. 3C). The operation lasted 5 hours and 30 minutes with intraoperative bleeding of 800 mL. Postoperative treatment included inhibiting pancreatic enzyme secretion, nutritional support, antiinfection and symptomatic treatment was strengthened. There were no complications such as pancreatic and bile leakage. The pathological findings were that a nodular mass 11.5 × 7.5 × 6.0 cm in size was seen in the head and body of the pancreas. Most of the sections were solid, grayish yellow and grayish white, and a few were cystic and grayish white. The diameter of the cyst was 1–3 mm. The boundary between the mass and surrounding pancreatic tissue was clear. Pathological analysis showed a PMSNN, containing both cystic and solid neuroendocrine components, with the collision type combination (Fig. 4A). In the cystic neoplasm, the glandular duct was expanded in a cyst shape, and lined with a single layer of cubical epithelium, interstitial fibrosis and sclerosis (Fig. 4B). In the neuroendocrine neoplasm, cells grew in a beam or pseudoglandular arrangement, with a consistent nuclear size, fine nuclear chromatin and eosinophilic or bichromatic cytoplasm, and the mitotic count was 0 per 10 high power fields (HPF) (Fig. 4C–D). Immunohistochemistry staining for chromogranin A in neuroendocrine neoplasm cells was positive (Fig. 4E). The Ki67 labeling index of the pancreatic NET was more than 5% (Fig. 4F). The pathological diagnosis was that PMSNN with the collision type combination and the well-differentiated grade 2 pancreatic NET. The patient recovered and was discharged on the 19th day after surgery.

At the 18 months follow-up visit, the patient had no abdominal pain, fever, or other discomfort. Her diet and liver function were normal, and no abnormal enhancement focus was found on abdominal enhanced CT.

## 3. Discussion

Keel SB et al^[6]^ first reported a case of pancreatic NET originating from a serous cystadenoma (SCN), and reports then gradually increased.^[7,8]^ The 2010 WHO classification of pancreatic tumors clearly puts forward the concept of mixed serous neuroendocrine tumor (MSNN), meaning that SCN and NEN occur simultaneously in the pancreas, and MSNN is listed as a new subtype of SCN.^[3,4]^ However, due to a lack of understanding of this tumor in clinical practice, it is often misdiagnosed and the diagnosis is missed.^[9]^

PMSNN is termed mixed tumors containing 2 components with different pathologies, PSCN and PanNET. Based on the distribution pattern of PSCN and PanNET, PMSNN is categorized into 4 subtypes^[3,4,10]^: (1) Diffuse subtype: The entire pancreas is occupied by numerous PSCN associated with one or more PanNET, which could be located in any region of the pancreas; (2) Mixed subtype: The 2 different tumors combined with each other in a mass cannot be distinctly divided; (3) Solitary subtype: The 2 different components coincide in the pancreas without any intermingling area; and (4) Collision subtype: The 2 different lesions are separated from each other in most parts, but with a partially intermixed or overlapping zone. PanNET is further graded on a scale of 1–3 based on mitotic rates and Ki67 proliferation index as follows: PanNET grade 1 (G1) with <2 mitoses/10 HPF and a <3% Ki67 index; PanNET grade 2 (G2) with 2–20 mitoses/10 HPF and a 3–20% Ki67 index; and PanNET grade 3 (G3) with >20 mitoses/10 HPF and a > 20% Ki67 index. Accordingly, after reviewing the pathological images of the previous case reports of PMSNN, all the PanNET components diagnosed as “low-grade/well-differentiated malignant NET”or “well-differentiated NEC” should be reclassified as well-differentiated PanNET rather than a poorly differentiated PanNEC. Therefore, our case belongs to the Collision subtype of PMSNN and the well-differentiated grade 2 PanNET.

PMSNN is an extremely rare disease and is often associated with VHL syndrome, which is a rare and autosomal dominant disease with a tendency to become cancerous. The incidence of VHL syndrome is approximately 1/36,000 to 1/45,500. Approximately 60% of patients with VHL syndrome have pancreatic lesions,^[11–13]^ which can manifest as simple cysts, SCN and NEN. The malignant transformation rate of SCN is low,^[14]^ and asymptomatic patients do not need to be treated. Most NEN have no function but have a tendency for malignant transformation,^[15]^ which requires careful treatment, including regular follow-up, surgical treatment and drug treatment. At present, there are no special guidelines for pancreatic lesions in VHL syndrome.^[16]^ Before hospitalization, although this patient had a history of “left oophorectomy for ovarian borderline mucinous cystadenoma canceration (well differentiated mucinous adenocarcinoma)” 11 months previously, no lesions in other organs were found during preoperative examination, and no abnormalities in other organs were found during the 1 year postoperative follow-up period. In addition, VHL gene detection was normal, therefore VHL syndrome was excluded.

Since this case belongs to a simple PMSNN unrelated to VHL syndrome, and the NET component is a well-differentiated G2 PanNET, it is necessary to further explore the treatment principles of nonfunctional PanNET. The Chinese guidelines for the diagnosis and treatment of pancreatic neuroendocrine neoplasms (2020)^[15]^ suggested that the surgical strategy of nonfunctional PanNET usually depends on the tumor size and pathological grade. For the patients with G1 and G2 PanNET whose maximum tumor diameter is <2 cm, imaging follow-up can be carried out every 6 to 12 months on the premise of full communication with patients and their families; for those with G2 PanNET, surgical treatment should be considered. In the follow-up period, if patients have significant tumor growth (usually by more than 20%), and show evidence of regional lymph node metastasis or signs of local invasion, and pancreatic duct dilatation or obstructive jaundice, immediate surgery should be carried out. The choice of surgical method depends on the general condition of the patient, the location of the tumor, and the number of tumors. For nonfunctional G1 and G2 PanNET with a small maximum diameter, local tumor resection and minimally invasive surgery are recommended, which have advantages in reducing intraoperative bleeding, shortening operation time and protecting pancreatic secretory function, and the long-term prognosis of patients is not statistically different from that of patients receiving conventional pancreatic resection.^[17]^ For PanNET located in the head and uncinate process of the pancreas, or adjacent to the main pancreatic duct, or multiple tumors in the pancreas, it is generally recommended to perform conventional pancreatectomy and dissect the peripancreatic lymph nodes.

For nonfunctional PanNET with a maximum diameter ≥ 2 cm, conventional pancreatectomy and regional lymph node dissection are recommended.^[18]^ For PanNET of the pancreatic head and uncinate process, pancreaticoduodenectomy is recommended, and pancreatic head resection with organ preservation can also be performed under specific conditions according to the maximum diameter and extent of tumor invasion. Segmental pancreatectomy is feasible for tumors in the body of pancreas. For tumors in the body and tail of the pancreas, distal pancreatectomy (including combined splenectomy) is recommended.

Pancreatic cancer easily expands into the retroperitoneal space, involving the main arteries and veins around the pancreas. This is of great significance in determining whether the pancreatic cancer can be surgically removed and for evaluating the prognosis of patients. Therefore, it is very important to accurately evaluate the main blood vessels of the pancreas during preoperative imaging.^[19]^ Abdominal multi-slice spiral CT (MSCT) enhanced scanning, combined with 3D reconstruction technology can obtain images of anatomical structure around the pancreas, which can accurately reflect the pancreatic mass itself and the main blood vessels around the pancreas.^[20]^ In our patient, abdominal MSCT was used to enhance scanning, combined with 3D reconstruction, including MIP and 3D visualization reconstruction, to examine the main blood vessels around the pancreas. Abdominal enhanced CT images showed the enhanced mass with a nonenhanced cyst involving the head and body of the pancreas, which invaded important surrounding blood vessels. Coronal surface MIP images revealed that the soft tissue mass invaded the common hepatic artery, superior mesenteric artery, inferior mesenteric artery, portal vein, and superior mesenteric vein. However, 3D visualization reconstruction images showed that the vessel walls of the portal vein, superior mesenteric vein, and superior mesenteric artery were intact. Radical pancreatoduodenectomy confirmed that the main blood vessels around the pancreas were compressed or even wrapped by the mass, but the blood vessel walls were not penetrated.

Therefore, with regard to PMSNN invading peripancreatic vessels, it is worthwhile operating on the basis of preoperative abdominal CT 3D visualization reconstruction evaluation. Even if abdominal enhanced CT shows that important peripancreatic vessels are invaded, this is not an absolute contraindication. In addition, even if there is a history of left ovarian borderline mucinous cystadenoma canceration, it is not necessarily metastatic cancer. If metastatic cancer is present, but not extensive metastasis, surgical exploration in the presence of simple vascular invasion is still possible. If there is vascular compression, surgery is of great significance. If the blood vessels are infiltrated, wrapped and fused, replacement with artificial blood vessels and allogeneic blood vessels may prolong the life of patients.

The prognosis of PMSNN is associated with the classification, grading and staging of tumors. The Chinese guidelines for the diagnosis and treatment of pancreatic neuroendocrine neoplasms (2020)^[15]^ suggested that all PanNET patients should be followed up regularly. For low-risk PanNET patients who did not receive surgical treatment, the follow-up is to monitor the progress of primary tumor and possible tumor metastasis. For PanNET patients undergoing radical surgery, the follow-up is to exclude the tumor recurrence in situ and metachronous tumor metastasis.

## 4. Conclusion

In summary, this case demonstrates that surgical treatment of PMSNN with invasion of peripancreatic vessels can be successful. Preoperative abdominal CT 3D visualization reconstruction is helpful in determining the degree of invasion of important peripancreatic vessels, and plays a key role in formulating an accurate surgical plan and improving patient outcome.

## Author contributions

Conceptualization: Zongming Zhang, Limin Liu, Youwei Li.

Investigation: Zongming Zhang, Limin Liu, Youwei Li, Zhuo Liu, Chong Zhang, Yue Zhao, Mingwen Zhu, Baijiang Wan, Hai Deng, Xiyuan Xie, Kun Tian, Zhentian Guo, Haiyan Yang, Jiahong Liao, Hongyan Zhu, Lili Liu, Man Wang, Xiaoting Ma, Tiantian Liu, Niuniu Huang, Yujiao Gao, Jing Zhao, Fang Liao, Fengyuan Li, Xueting Wang, Ruijiao Yuan, Xinying Liu, Lidan Chang.

Funding acquisition: Zongming Zhang.

Supervision: Zongming Zhang.

Writing—original draft: Zongming Zhang, Limin Liu, Youwei Li.

Writing—review & editing: Zongming Zhang.
